# Emergency Resection for Perforated Small Bowel Metastasis from Primary Lung Cancer During Systemic Therapy: A Case Report

**DOI:** 10.70352/scrj.cr.26-0567

**Published:** 2026-11-03

**Authors:** Yoichi Miyaoka, Shingo Shimada, Yoshifumi Iwaguchi, Yoshikazu Ganchiku, Tomoya Saito, Ryoji Yokoyama, Daichi Hanaoka, Akinobu Taketomi

**Affiliations:** 1 Department of Surgery, JA Hokkaido Koseiren Abashiri-Kosei General Hospital, Abashiri, Hokkaido, Japan; 2 Department of Surgery, Otaru General Hospital, Otaru, Hokkaido, Japan; 3 Department of Diagnostic Surgical Pathology, JA Hokkaido Koseiren Sapporo Kosei General Hospital, Sapporo, Hokkaido, Japan; 4 Department of Gastroenterological Surgery I, Faculty of Medicine and Graduate School of Medicine, Hokkaido University, Sapporo, Hokkaido, Japan

**Keywords:** lung cancer, small bowel metastasis, intestinal perforation, emergency surgery, immune checkpoint inhibitor

## Abstract

**INTRODUCTION:**

Small bowel metastases from lung cancer may remain clinically occult until serious complications, including obstruction, bleeding, and perforation, occur. We report a case of perforated jejunal metastasis with a synchronous ileal metastasis at high risk of obstruction, identified during emergency surgery while several previously documented extraintestinal lesions had decreased in size.

**CASE PRESENTATION:**

A 75-year-old woman with metastatic non-small cell lung carcinoma favoring adenocarcinoma had a programmed death-ligand 1 tumor proportion score of 95% and no targetable alteration on the companion-diagnostic panel. Pembrolizumab monotherapy (200 mg intravenously every 3 weeks) was administered for 4 cycles; no antiangiogenic agent had been administered concurrently or previously. Eleven days after the 4th dose, she developed acute abdominal pain. CT showed focal small bowel wall thickening, adjacent extraluminal air, diffuse bowel edema, and ascites. Emergency surgery revealed a perforated jejunal tumor approximately 20 cm distal to the ligament of Treitz. Systematic examination of the entire small bowel identified a 2nd, nearly circumferential ileal tumor approximately 15 cm proximal to the terminal ileum with marked luminal narrowing; both lesions were resected. Histopathological examination showed metastatic poorly differentiated adenocarcinoma of pulmonary origin. Neither tumor necrosis nor a histological treatment effect was identified, and the non-neoplastic mucosa showed no findings suggestive of immune checkpoint inhibitor-related enteritis. The patient was discharged on POD 19. Pembrolizumab was not resumed. PET-CT on POD 30 demonstrated widespread disease progression, and the patient transitioned to best supportive care. She died on POD 61 without recurrent gastrointestinal perforation, obstruction, or bleeding.

**CONCLUSIONS:**

Clinically occult small bowel metastases may present with perforation or impending obstruction during systemic therapy even while previously documented lesions at other sites decrease in size. Because the intestinal lesions were not identifiable before treatment, true discordant progression could not be established. Histopathological findings supported direct tumor-related perforation rather than treatment-induced tumor necrosis or immune-mediated enteritis. Emergency source control and systematic examination of the entire small bowel may identify additional lesions that alter operative management.

## Abbreviations

ALK
: anaplastic lymphoma kinase
CDX2
: caudal type homeobox 2
CK
: cytokeratin
ECOG
: Eastern Cooperative Oncology Group
EGFR
: epidermal growth factor receptor
NRAS
: neuroblastoma RAS viral oncogene homolog
PD-L1
: programmed death-ligand 1
TTF-1
: thyroid transcription factor-1

## INTRODUCTION

Primary lung cancer commonly metastasizes to the brain, liver, adrenal glands, and bone, whereas gastrointestinal metastasis is relatively uncommon in clinical practice. However, gastrointestinal involvement may be underrecognized, particularly in patients with advanced disease.^1–4)^ Small bowel metastases are clinically important because they may cause obstruction, bleeding, intussusception, and perforation.^4–6)^ Perforation may rapidly progress to generalized peritonitis and requires urgent surgical management.^5–7)^ Multiple small bowel metastatic lesions have also been reported, creating a risk of additional intestinal complications after treatment of the presenting lesion.^8)^

We report a patient with a perforated jejunal metastasis from lung cancer and a synchronous ileal metastasis with marked luminal narrowing detected during systematic examination of the entire small bowel. We focus on the systemic treatment history, retrospective imaging findings, pathological assessment of the perforation mechanism, and postoperative oncological outcome. This case report was prepared in accordance with the CAse REport (CARE) reporting guidelines.

## CASE PRESENTATION

A 75-year-old woman had been diagnosed with stage IVB squamous cell carcinoma of the lung with lumbar vertebral metastasis at another hospital in 2017. She received radiotherapy followed by pembrolizumab from December 2017 to September 2021. Thereafter, she was followed without clinically apparent recurrence. The original pathological specimens and pathology report from 2017 were unavailable for review.

In September 2025, routine follow-up CT revealed multiple new nodular lesions in the lungs that had not been observed previously. Transbronchial biopsy of a lesion in the left lingular segment revealed non-small cell carcinoma, favoring adenocarcinoma. Immunohistochemical staining was positive for TTF-1, partially positive for Napsin A, and negative for p40 and CK 5/6. The PD-L1 tumor proportion score was 95%. Molecular testing revealed no actionable driver alterations, including EGFR and ALK. An NRAS variant was detected but was not considered a targetable alteration under the companion-diagnostic panel.

Because the pulmonary lesions were multifocal, the exact T and N categories were not formally documented. Imaging also demonstrated metastases to the liver, right adrenal gland, bone, and pleura, and a pancreatic lesion was considered metastatic. The disease was therefore clinically diagnosed as stage IVB lung cancer. Her ECOG performance status was 1. Given the high PD-L1 expression and the absence of actionable driver alterations, pembrolizumab monotherapy was selected after discussion with the patient.

Pembrolizumab was administered at 200 mg intravenously every 3 weeks for 4 cycles. Eleven days after the 4th dose, the patient developed sudden generalized abdominal pain and was transported to our emergency department. She had no diarrhea or vomiting. On arrival, her temperature was 37.0°C, blood pressure was 133/70 mmHg, pulse rate was 106 beats/min, and oxygen saturation was 96% on room air. Physical examination revealed diffuse abdominal tenderness with rebound tenderness, most prominent in the right lower quadrant. Laboratory tests showed mild anemia, hypoalbuminemia, and mildly elevated inflammatory markers, without an elevated lactate level (**Table 1**).

**Table 1:** Laboratory findings on admission

Parameter	Value
White blood cell count	6.2 × 10^3^/μL
Hemoglobin	9.4 g/dL
Platelet count	445 × 10^3^/μL
Total protein	5.8 g/dL
Albumin	3.3 g/dL
Total bilirubin	0.5 mg/dL
Aspartate aminotransferase	18 U/L
Alanine aminotransferase	7 U/L
Lactate dehydrogenase	326 U/L
Alkaline phosphatase	106 U/L
γ-Glutamyl transpeptidase	11 U/L
Blood urea nitrogen	12.9 mg/dL
Creatinine	0.53 mg/dL
Sodium	145 mmol/L
Potassium	3.5 mmol/L
Chloride	109 mmol/L
C-reactive protein	1.73 mg/dL
Prothrombin time	112%
PT-INR	0.94
Activated partial thromboplastin time	27.3 s
D-dimer	7.4 μg/mL
Procalcitonin	0.35 ng/mL
Lactate	1.35 mmol/L

PT-INR, prothrombin time–international normalized ratio

No scheduled response-assessment CT had been performed between the initiation of pembrolizumab and the onset of bowel perforation; therefore, the emergency CT was the 1st post-treatment CT. Compared with the CT obtained at treatment initiation, the previously documented pulmonary and pleural lesions, liver metastasis, right adrenal lesion, and pancreatic lesion had decreased in size, and the left pleural effusion had also decreased. The emergency CT showed diffuse small bowel wall edema, intraluminal fluid accumulation, ascites, and inflammatory changes. Gastrointestinal perforation was suspected based on focal small bowel wall thickening and adjacent extraluminal air (**Fig. 1B**–**1D**). Small bowel metastases could not be clearly identified preoperatively.

**Fig. 1:**
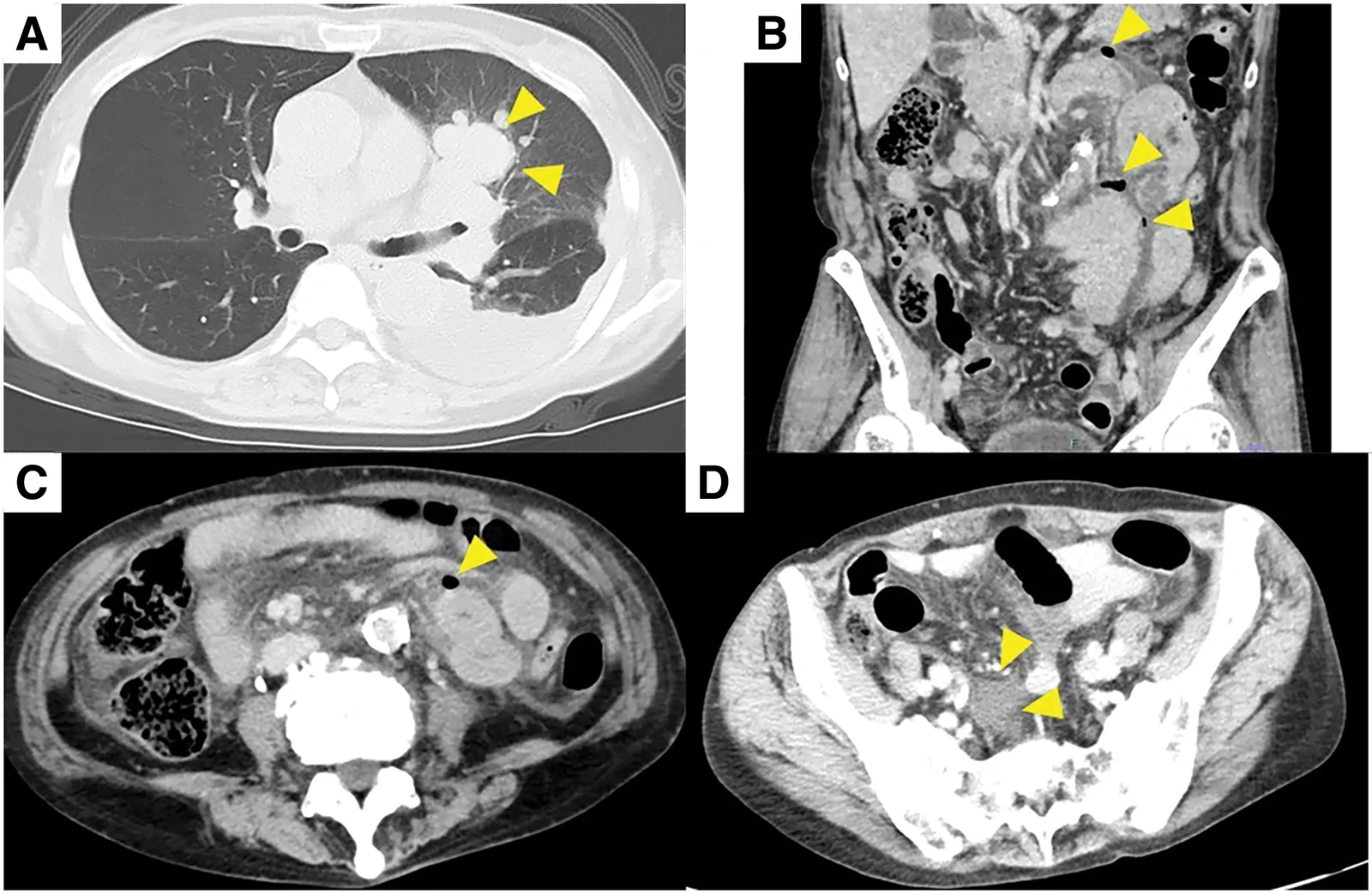
Preoperative CT findings. (**A**) Chest CT showing the left lingular lung lesion (arrowheads). (**B**) Coronal abdominal CT showing focal small bowel wall thickening; the arrowheads indicate adjacent extraluminal air. (**C**) Axial CT showing extraluminal air adjacent to the affected small bowel (arrowhead). (**D**) Axial CT showing edematous small bowel loops and ascites; the arrowheads indicate ascites between the bowel loops.

Emergency surgery was performed. Diagnostic laparoscopy revealed purulent ascites throughout the abdominal cavity and diffuse erythematous and edematous changes of the small bowel. A perforated tumorous lesion was identified in the jejunum (**Fig. 2A**–**2C**). After extension of the umbilical incision and exteriorization of the small bowel, a hard tumorous lesion approximately 20 cm distal to the ligament of Treitz was found to have perforated centrally, with leakage of intestinal contents (**Fig. 2D**). Segmental jejunal resection was performed.

**Fig. 2:**
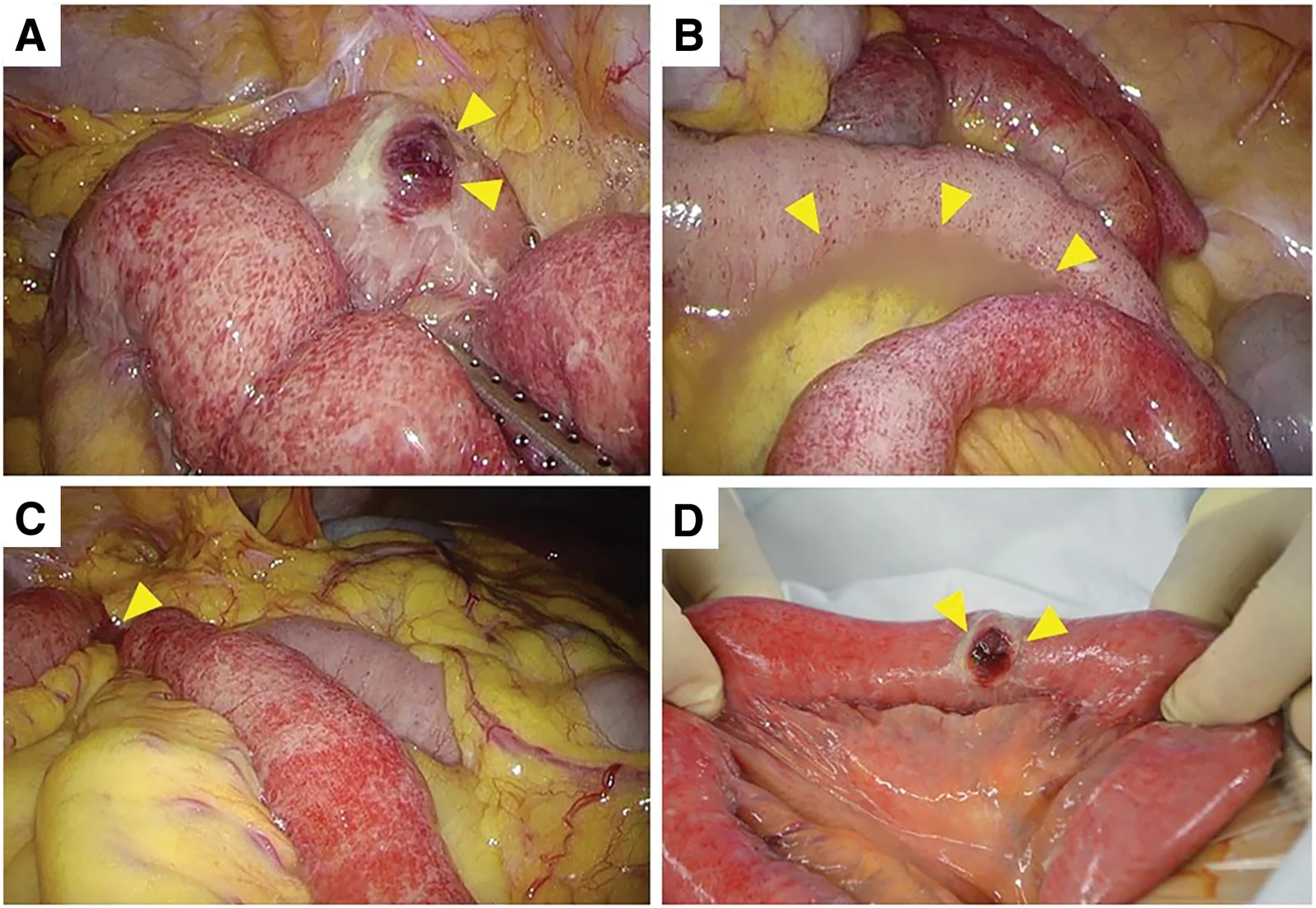
Intraoperative findings. (**A**) Diagnostic laparoscopy revealed a perforated jejunal lesion; the arrowheads indicate the perforation site. (**B**) Purulent ascites was present in the abdominal cavity; the arrowheads indicate purulent ascites between the bowel loops. (**C**) Diffuse inflammatory changes with erythematous and edematous small bowel loops were observed laparoscopically; the arrowhead indicates an affected small bowel loop. (**D**) The perforated jejunal lesion was confirmed after exteriorization of the bowel; the arrowheads indicate the perforation site.

The entire small bowel was then systematically examined from the ligament of Treitz to the ileocecal valve. A 2nd, nearly circumferential tumorous lesion was identified approximately 15 cm proximal to the terminal ileum. The lesion caused marked luminal narrowing and was considered to be at high risk of subsequent obstruction; therefore, additional segmental ileal resection was performed. No other small bowel lesions were identified. Functional end-to-end anastomoses were created for both resected segments. The operative time was 1h 56 min, blood loss was 50 mL, and no transfusion was required.

After surgery, the pretreatment and emergency CT images were retrospectively reviewed with reference to the locations of the resected jejunal and ileal lesions. On the pretreatment CT, no definite focal abnormality corresponding to either lesion could be identified. On retrospective review of the emergency CT, the perforated jejunal lesion could be localized based on focal wall thickening and adjacent extraluminal air. In contrast, the synchronous ileal lesion remained indistinguishable from the diffuse small bowel edema, ascites, and inflammatory changes. No PET-CT had been performed before the perforation.

Macroscopic examination of the resected specimens showed an ulcerative perforated tumor in the jejunum and a nearly circumferential ulcerative tumor with marked luminal narrowing but without perforation in the ileum (**Fig. 3A**, **3B**). The jejunal and ileal lesions measured 30 × 15 mm and 37 × 22 mm, respectively.

**Fig. 3:**
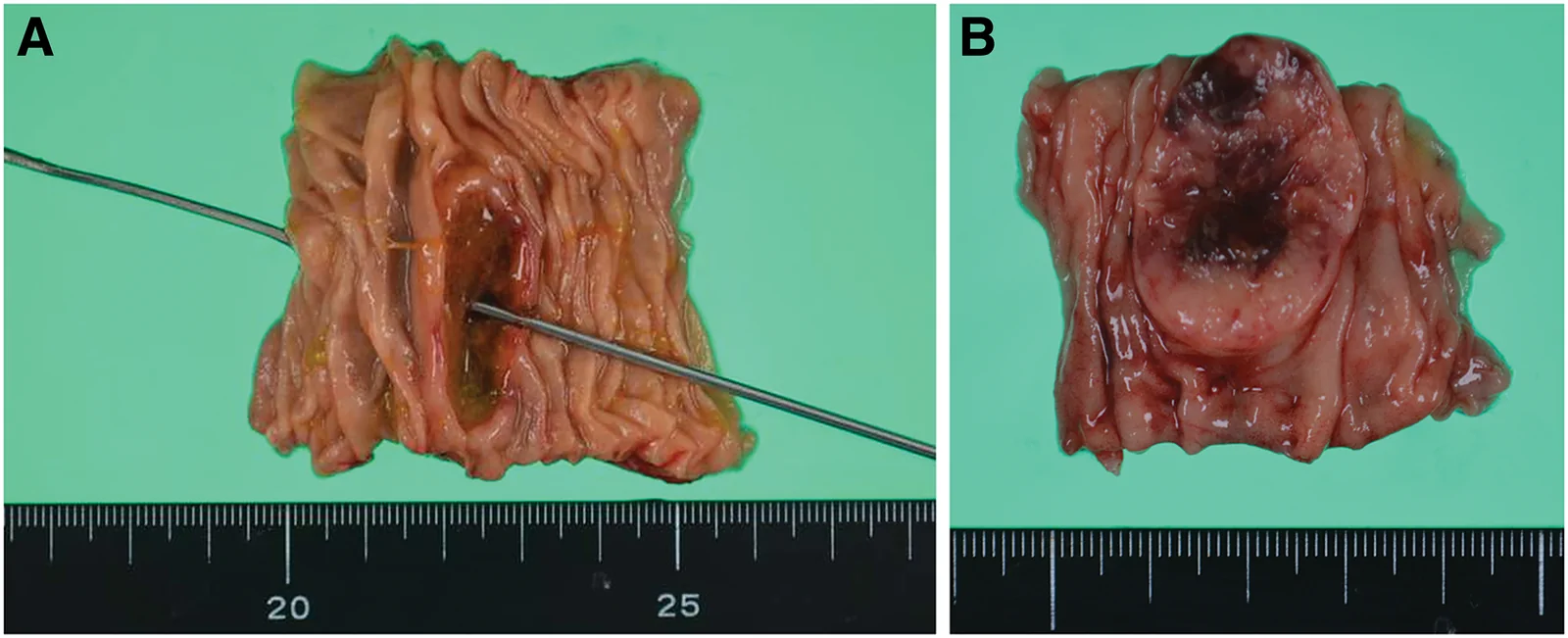
Macroscopic appearances of the resected jejunal and ileal specimens. (**A**) The jejunal specimen shows an ulcerative, perforated tumor. (**B**) The ileal specimen shows a nearly circumferential ulcerative tumor without perforation, with marked luminal narrowing and a high risk of obstruction.

Histopathological examination showed poorly differentiated adenocarcinoma in both lesions, with predominantly submucosal solid growth and mucosal ulceration (**Fig. 4A**, **4B**). In the jejunal lesion, the tumor was exposed at the serosal surface, with surrounding neutrophilic infiltration and fibrin deposition, supporting direct tumor-related perforation.

**Fig. 4:**
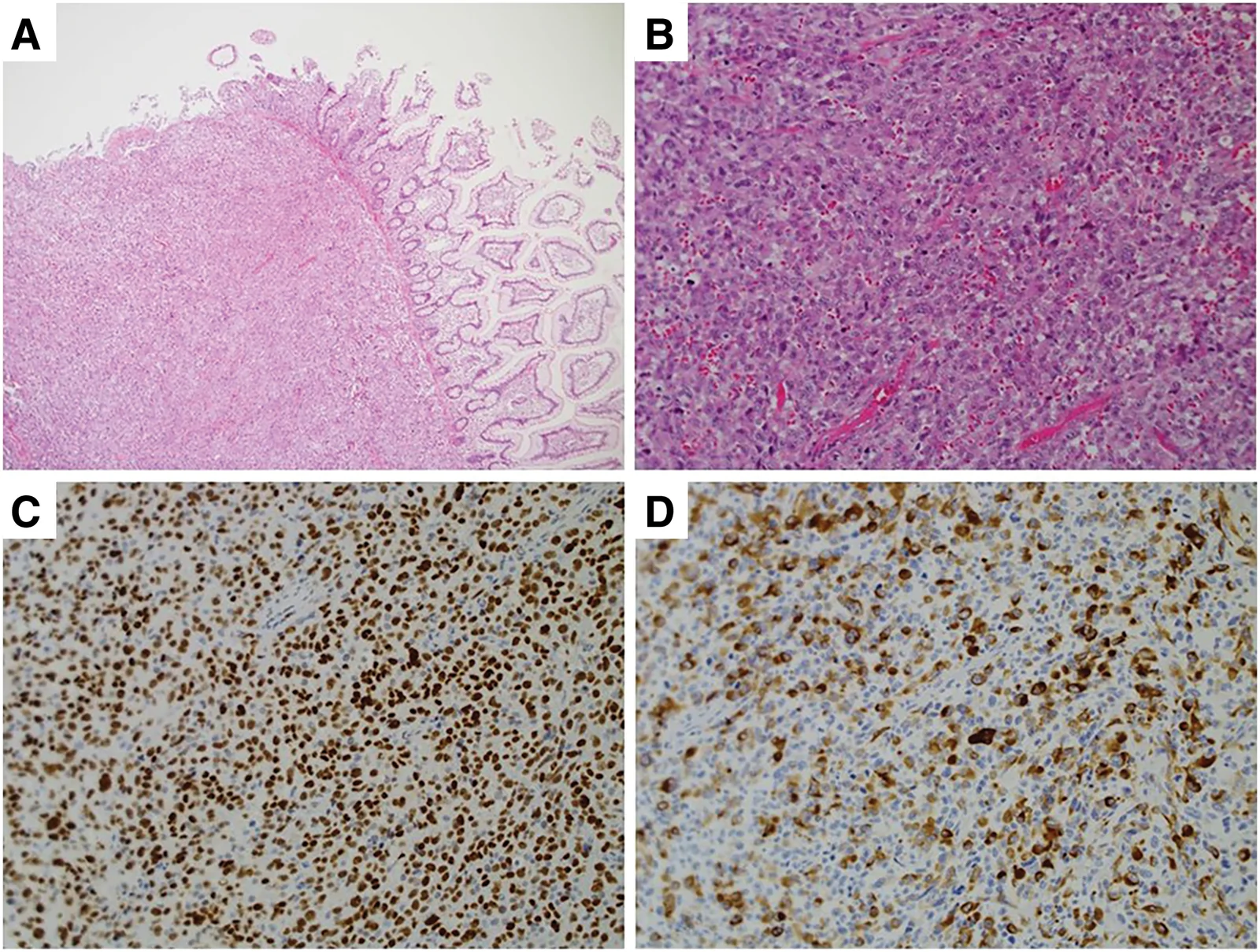
Histopathological and immunohistochemical findings. (**A**) Low-power H&E staining showing submucosal-predominant tumor proliferation adjacent to the intestinal mucosa. (**B**) High-power view showing poorly differentiated adenocarcinoma cells with marked nuclear atypia. (**C**) Immunohistochemical staining for TTF-1 showing nuclear positivity in the tumor cells. (**D**) Immunohistochemical staining for CK7 showing focal positivity. Original magnification: (**A**) ×40; (**B**) ×200. CK7, cytokeratin 7; H&E, hematoxylin and eosin; TTF-1, thyroid transcription factor-1

Immunohistochemically, the intestinal tumor cells were positive for TTF-1 (**Fig. 4C**), focally positive for CK7 (**Fig. 4D**), and negative for Napsin A, CDX2, CK20, p40, and CK5/6. The morphological and immunohistochemical findings were consistent with those of the 2025 lung biopsy, supporting the diagnosis of small bowel metastases from lung cancer. Neither tumor necrosis nor a histological treatment effect was identified. In addition, the non-neoplastic intestinal mucosa showed no findings suggestive of immune checkpoint inhibitor-related enteritis.

The postoperative course was uneventful. Oral intake was resumed on POD 4, and the patient was discharged on POD 19. Pembrolizumab was not resumed. PET-CT performed on POD 30 demonstrated widespread disease progression involving multiple organs, including the lungs, pleura, and bone. No further systemic therapy was administered, and the patient transitioned to best supportive care. She died on POD 61. No recurrent gastrointestinal perforation, obstruction, bleeding, or reoperation occurred before death. The clinical course is summarized in **Table 2**.

**Table 2:** Clinical timeline

Time point	Clinical event
2017–September 2021	Stage IVB lung squamous cell carcinoma with lumbar vertebral metastasis was treated at another hospital with radiotherapy and pembrolizumab. No clinically apparent recurrence was subsequently documented.
September 2025	Routine follow-up CT revealed multiple new pulmonary nodules. Transbronchial biopsy of a left lingular lesion showed non-small cell carcinoma favoring adenocarcinoma. The programmed death-ligand 1 tumor proportion score was 95%. No actionable driver alteration was identified. ECOG performance status was 1.
October–December 2025	Pembrolizumab 200 mg intravenously every 3 weeks was administered for 4 cycles.
December 22, 2025	Eleven days after the fourth pembrolizumab dose, sudden abdominal pain developed. CT showed small bowel perforation. Emergency surgery identified a perforated jejunal metastasis and a synchronous nearly circumferential ileal metastasis with marked luminal narrowing; both lesions were resected.
POD 4	Oral intake was resumed.
POD 19	The patient was discharged without postoperative complications.
POD 30	PET-CT demonstrated widespread disease progression. Pembrolizumab was not resumed, and the patient transitioned to best supportive care.
POD 61	The patient died without recurrent gastrointestinal perforation, obstruction, or bleeding.

ECOG, Eastern Cooperative Oncology Group

## DISCUSSION

Gastrointestinal metastasis from lung cancer is uncommon in routine clinical practice but is likely underrecognized in patients with advanced disease. Previous clinical studies reported gastrointestinal metastasis in 0.2%–1.7% of patients with lung cancer, whereas autopsy studies reported higher rates of 4.7%–14.0%.^3,4)^ Once symptomatic, small bowel metastases may cause obstruction, bleeding, intussusception, or perforation.^4–6)^ Perforation is particularly serious because it may lead rapidly to generalized peritonitis or sepsis and generally requires emergency source control.^5,7)^

Preoperative identification of small bowel metastases remains difficult when they are clinically occult. Reported CT findings include focal or segmental wall thickening, an intraluminal mass, intussusception, surrounding inflammatory changes, and perforation.^6,9)^ In this patient, no definite intestinal lesion was identified on retrospective review of the available pre-treatment CT images. On the emergency CT, diffuse bowel edema, intraluminal fluid, ascites, and inflammatory changes obscured focal abnormalities; the perforated jejunal segment was suggested by adjacent extraluminal air, but the synchronous ileal lesion remained unrecognizable. In addition, no interval response-assessment CT had been performed. Consequently, the temporal behavior and treatment response of the intestinal lesions could not be determined.

Surgery for patients with disseminated lung cancer is generally palliative rather than curative. Nevertheless, perforation with generalized peritonitis requires urgent source control, and resection may provide symptom relief and short-term recovery.^7,8,10,11)^ Systematic examination of the small bowel is standard surgical practice during an operation for intestinal perforation; therefore, the examination itself is not a novel technique. The clinical significance in this case was that examination identified a 2nd, radiographically occult, nearly circumferential lesion with marked luminal narrowing, which changed the operative plan and led to simultaneous resection. A Japanese multicenter analysis including small bowel metastases from various primary cancers reported multiple small bowel metastatic lesions in 11% of patients,^8)^ supporting careful examination of the entire small bowel when the patient’s condition permits.

In this patient, clinically significant intestinal metastases became apparent while several previously documented pulmonary and extraintestinal lesions had decreased in size. However, because neither intestinal lesion had been clearly identified before treatment and no interval response-assessment CT was available, true discordant progression cannot be established. Thus, radiographic improvement of known disease does not exclude a clinically occult intestinal metastasis capable of perforation or obstruction.

Possible mechanisms of perforation include direct transmural tumor involvement, treatment-associated tumor necrosis, and treatment-related intestinal inflammation. Small bowel perforation during pembrolizumab treatment has been reported,^12)^ but temporal association alone does not establish causality. In this patient, no antiangiogenic agent had been administered. Histological examination demonstrated mucosal ulceration, tumor exposure at the serosal surface, neutrophilic infiltration, and fibrin deposition. Conversely, neither tumor necrosis nor a histological treatment effect was identified, and the non-neoplastic mucosa showed no findings suggestive of immune checkpoint inhibitor-related enteritis. These findings most strongly support direct tumor-related perforation rather than treatment-induced tumor necrosis or immune-mediated enteritis. Accordingly, the event is best described as perforation of a metastatic lesion occurring during systemic therapy, rather than as a confirmed adverse effect of pembrolizumab.

Pathological comparison was particularly important because the patient had a remote history of lung squamous cell carcinoma diagnosed at another hospital. The 2025 lung biopsy showed non-small cell carcinoma favoring adenocarcinoma, with TTF-1 positivity, partial Napsin A positivity, and negativity for p40 and CK5/6. Both intestinal lesions showed poorly differentiated adenocarcinoma with TTF-1 positivity and negativity for p40 and CK5/6, supporting pulmonary origin and a relationship to the 2025 lung lesion. Because the original 2017 pathological material was unavailable, the relationship between the historical and 2025 pulmonary malignancies could not be definitively established.

This case provides 3 practical lessons. First, an occult small bowel metastasis should remain in the differential diagnosis when a patient with lung cancer develops acute abdominal symptoms during systemic therapy, even while previously documented lesions are decreasing in size. Second, when emergency surgery is required, systematic examination of the entire small bowel may identify an additional lesion that changes the operative plan; in this case, it enabled simultaneous resection of a 2nd lesion considered at high risk of obstruction. Third, pathological assessment of tumor necrosis, treatment effect, and the non-neoplastic mucosa can help distinguish direct tumor-related perforation from a treatment-related event. The principal limitations are that the intestinal lesions were not identifiable on prior imaging, no interval response-assessment CT was available, and the patient’s survival after surgery was limited by rapid systemic progression.

## CONCLUSIONS

Small bowel metastases from lung cancer may remain radiographically occult and present abruptly with perforation or impending obstruction during systemic therapy. In this patient, pathological findings supported direct tumor-related perforation rather than treatment-induced tumor necrosis or immune-mediated enteritis. Emergency resection achieved source control, and systematic examination of the entire small bowel identified a 2nd lesion that changed the operative plan. Because the intestinal lesions were not identifiable before treatment, true discordant progression could not be established.

## DECLARATIONS

### Availability of data and materials

All data supporting the conclusions of this case report are included within the article. Additional de-identified data are available from the corresponding author on reasonable request, subject to patient privacy and institutional requirements.
